# Virtual Reality Intervention to Help Improve Motor Function in Patients Undergoing Rehabilitation for Cerebral Palsy, Parkinson’s Disease, or Stroke: A Systematic Review of Randomized Controlled Trials

**DOI:** 10.7759/cureus.16763

**Published:** 2021-07-30

**Authors:** Jashvini Amirthalingam, Gokul Paidi, Khadija Alshowaikh, Anuruddhika Iroshani Jayarathna, Divya Bala Anthony Manisha R Salibindla, Katarzyna Karpinska-Leydier, Huseyin Ekin Ergin

**Affiliations:** 1 General Medicine, California Institute of Behavioral Neurosciences & Psychology, Fairfield, USA; 2 Internal Medicine, California Institute of Behavioral Neurosciences & Psychology, Fairfield, USA; 3 Obstetrics and Gynecology, California Institute of Behavioral Neurosciences & Psychology, Fairfield, USA; 4 Neurology, California Institute of Behavioral Neurosciences & Psychology, Fairfield, USA; 5 General Practice, California Institute of Behavioral Neurosciences & Psychology, Fairfield, USA

**Keywords:** cerebral palsy, stroke, parkinson's disease, rehabilitation, virtual reality, motor function, neural plasticity, visual feedback, gait, posture

## Abstract

There are many successful interventions in medicine, especially in neurology and rehabilitation. The neurosciences represent an area of medicine with tremendous recent research innovations, one of which is virtual reality. This paper aims to discover the powerful relationship between virtual reality and rehabilitation. We assessed the effectiveness of virtual reality-based rehabilitation compared to conventional rehabilitation on motor function recovery of three patient groups: patients with a diagnosis of cerebral palsy, Parkinson's disease, or stroke. We conducted a systematic review using PubMed and included only articles that were randomized controlled trials that were published in the last five years. We used a general search in combination with a more focused Medical Subject Headings (MeSH) search. After thorough assessment and risk of bias evaluation using the Cochrane risk of bias tool, we included thirteen studies in this review. The majority of the clinical trials showed a statistically significant effect for improved motor function. More specifically, improvements in upper extremity motor function, gait, and balance in patients diagnosed with stroke were seen. Similarly, when evaluating patients with Parkinson's disease, improved gait and posture were also seen. When it came to cerebral palsy, however, there were no significant differences between the experimental group and the control. The level of improvement in motor function with a virtual reality intervention was striking, particularly since a few studies demonstrated sustained motor improvement a few months post-trial as well. Virtual reality-based rehabilitation has promising results for adult patients diagnosed with stroke or Parkinson's disease. For pediatric patients, on the other hand, a larger number of clinical trials would still need to be conducted to validate if virtual reality interventions have the capability of providing improved motor function recovery.

## Introduction and background

"An advanced imaginal system, in other words, a modern form of imagery that is just as effective as reality in inducing emotions and expressions - this is virtual reality." [1-3]. It may seem that virtual reality (VR) applications are the newest and latest form of technology based on recent scientific research; however, in reality, VR is not as new as one would expect. When it comes to the history of VR, the concept originated in the mid-1960s [4-5]. VR was, at that time, described as an interface where a user would be able to experience a virtual environment using perception, presence, and immersion [4-15]. VR is so unique in that it allows its users to interact dynamically and in real-time with that specially designed virtual interface. Initially, VR applications were used in technology-related fields such as graphics and video games [1]. In recent years, just like how a neural network is formed from axial branches and collaterals, the use of VR has extended tremendously into many disciplines, having a very strong and powerful impact in the medical sciences. 

Currently, numerous randomized clinical trials are being conducted to evaluate the effectiveness of VR as a therapeutic tool in patients diagnosed with neurological conditions. More specifically, the effectiveness of VR as a tool for therapeutic outcomes in gait, posture, and balance for patients recently diagnosed with stroke has been studied [16]. Furthermore, extending onto another branch of behavioral science, VR technologies have been used as a diagnostic tool in the field of pediatric psychiatry, especially with patients diagnosed with attention deficit hyperactivity disorder (ADHD) and autism spectrum disorder [1]. The extension of VR into adult psychiatry has also shown favorable outcomes when it comes to exposure therapy, eating disorders, anxiety disorders, and pain management [1]. Not surprisingly, the versatility of VR applications can be seen with its use in patients diagnosed with Chronic Obstructive Pulmonary Disease (COPD) for therapeutic recovery and as a fitness tool [17].

It has been noted that recent clinical trials are examining the effectiveness of the Nintendo Wii™ (Foxconn, New Taipei, Taiwan) console as a virtual reality application [18]. For example, there is a new randomized controlled trial being conducted using a Nintendo Wii-based game therapy for women diagnosed with mixed urinary incontinence [19]. This new study demonstrates how VR can be used to help diagnose and treat conditions across many medical specialties such as obstetrics and gynecology and not only limited to neurology or rehabilitation. To add, VR applications are also currently widely being used in surgical gynecology, for example, in medical education for VR robotic surgery simulation [20]. 

When it comes to VR as a tool in rehabilitation, it appears to be that many clinical trials have focused on neurological patients that are post-stroke; however, not much research has been conducted on patients diagnosed with Parkinson's disease or other neurological illnesses such as spinal injury. Furthermore, it is noticed that the majority of the clinical trials were done on adult patients when compared to pediatric patients. While there are a few articles on the concept of VR in pain management as a distractor in pediatrics, there aren't as many research studies examining the effectiveness of VR interventions in patients diagnosed with cerebral palsy or other pediatric neurological illnesses. This systematic review aims to evaluate the effectiveness of randomized clinical trials that have used VR in rehabilitation for motor function recovery in patients diagnosed with either stroke, Parkinson's disease, or cerebral palsy.

## Review

Methods

Protocol

This systematic review was performed according to the guidelines in the Preferred Reporting Items for Systematic Review and Meta-Analysis (PRISMA) [21]

Inclusion/Exclusion Criteria

The literature search for this systematic review was conducted to determine the effectiveness of a virtual reality intervention in patients undergoing neurological rehabilitation. The criteria used to search for eligible studies included the following: (1) pediatric, adult, or geriatric patients in rehabilitation therapy; (2) patients that were diagnosed with either cerebral palsy, Parkinson's disease, or stroke; and (3) virtual reality therapy as a sole therapy or as an adjunct therapy to an existing rehabilitation plan. The studies that reported other medical illnesses or conditions were excluded as they were outside the scope of this study. Only randomized controlled trials with articles published in English from the past five years (2016-2021) were included in this study. Systematic reviews, grey literature, books, documents, meta-analysis, clinical trials, and reviews were excluded. 

Search Strategy

A keyword-specific search of the databases PubMed® [22] and ScienceDirect [23] was conducted on April 30, 2021. The search for relevant studies using a generic keyword ("virtual reality") was done and populated 13,939 articles. The topic-specific Medical Subject Headings (MeSH) keywords "virtual reality," "Parkinson's disease," "stroke," "cerebral palsy," "rehabilitation," "neurological rehabilitation," "virtual reality exposure therapy," and "motor skills," were used in various combinations using Boolean operators like "OR" and "AND," and 1,096 relevant articles were identified. A search in ScienceDirect with the keywords "virtual reality," "cerebral palsy," and "randomized controlled trial" was performed and resulted in 437 articles with a final total of 15,472 articles. The inclusion/exclusion criteria were used, and research articles from January 2016 to April 2021 were obtained. The searched articles were managed using EndNote™ 20 (Clarivate Analytics, Philadelphia, Pennsylvania) and Microsoft Word (Microsoft, One Microsoft Way Redmond, Washington). Duplicates were removed using EndNote. Table 1 and Table 2 illustrate the results of the general search and more focused MeSH search strategy, along with the appropriate filters. Table 3 illustrates the results of the general keyword search in Science Direct. 

**Table 1:** Entire MeSH search Strategy (duplicates removed later) MeSH - Medical Subject Headings

MeSH terms	Total articles	2016-2021	Randomized controlled trials	Articles in English	Free full text
(("Treatment Outcome [Mesh]) OR ( "Rehabilitation"[Mesh] OR "Stroke Rehabilitation"[Mesh] OR "Neurological Rehabilitation"[Mesh] )) AND ( "Virtual Reality"[Mesh] OR "Virtual Reality Exposure Therapy"[Mesh] )	697	555	146	144	67
("Virtual Reality"[Mesh] OR "Virtual Reality Exposure Therapy"[Mesh]) AND ( "Stroke"[Mesh] OR "Stroke, Lacunar"[Mesh] OR "Hemorrhagic Stroke"[Mesh] OR "Embolic Stroke"[Mesh] OR "Thrombotic Stroke"[Mesh] OR "Ischemic Stroke"[Mesh] OR "Stroke Rehabilitation"[Mesh] )	239	201	50	49	30
"Motor Skills"[Mesh]) AND ( "Virtual Reality"[Mesh] OR "Virtual Reality Exposure Therapy"[Mesh] )	58	49	14	13	6
("Virtual Reality"[Mesh] OR "Virtual Reality Exposure Therapy"[Mesh]) AND "Parkinson Disease"[Mesh]	56	47	12	12	5
("Cerebral Palsy"[Mesh] OR "Cerebral Palsy, Ataxic, Autosomal Recessive" [Supplementary Concept] OR "Cerebral palsy, spastic, diplegic" [Supplementary Concept] OR "Cerebral Palsy, Spastic Quadriplegic, 2" [Supplementary Concept] OR "Cerebral Palsy, Spastic Quadriplegic, 1" [Supplementary Concept]) AND ( "Virtual Reality"[Mesh] OR "Virtual Reality Exposure Therapy"[Mesh] )	46	33	9	8	2

**Table 2:** General keyword search on Pubmed (duplicates removed later)

Regular keyword search on PubMed	Total articles	2016-2021	Randomized controlled trials	Articles in English	Free full text
Virtual reality	13939	7635	466	461	175

**Table 3:** ScienceDirect keyword search

Search term	Total articles	2016-2021	Research article	Randomized controlled trial	Free full text
virtual reality AND cerebral palsy AND randomized controlled trial	437	232	61	1	0

Results 

Search Outcome

**Figure 1:**
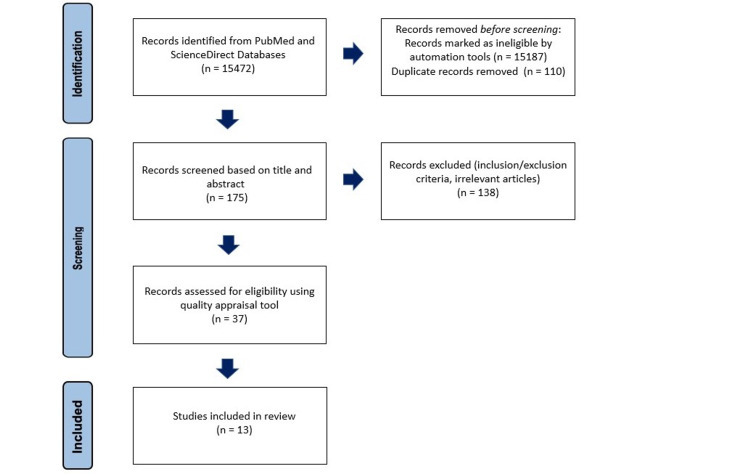
2020 Prisma flowchart illustrating the final articles included in this review n - number of articles

A cumulative total of 15,472 scientific articles were identified through a combination of a PubMed, MeSH search, and ScienceDirect. Out of this total, 15,187 articles were removed by filters or automation tools. For the duplicates, EndNote 20 was used, and 110 duplicates were removed, resulting in 175 articles. These articles were then screened based on title and abstract for eligibility. From 175 articles, 138 articles were excluded due to being irrelevant to the scientific question being studied, not meeting inclusion criteria, or satisfying exclusion criteria. After narrowing down the articles based on title and abstract, a total of 37 articles were selected for quality assessment. Since all of these studies comprised randomized clinical trials, the Cochrane risk-of-bias tool [24] was utilized. After the quality appraisal, a total of 13 articles were included in this systematic review. Figure 1 illustrates the 2020 PRISMA flow diagram and how the final 13 articles were derived after each exclusion step [21]. 

Data Extraction

To screen for title and abstract, the 175 articles were transported from Endnote 20 to Microsoft Word. The titles, abstracts, and free full-text articles were verified for eligibility by two reviewers independently, JA and GP. The components extracted from each article included author and year of publication, virtual reality intervention, target rehabilitation outcome, sample size, study design, results, and statistical significance. The randomized controlled trials accepted by one reviewer were also analyzed carefully by other reviewers for validity and accuracy. In case of disagreement, a mutual discussion was facilitated to arrive at a consensus. Articles with a low risk of bias were included in this systemic review. Table 4 shows the data table for the 13 articles included in this review [25-36]. 

**Table 4:** Data Table illustrating the study characteristics and findings for final articles included in this systematic review VERA - weaRable hAptic devices; RCT- randomized controlled trial; VR - virtual reality, GR - gesture recognition; BBS - Berg Balance Scale; FGA - Functional Gait Assessment; TUG - Timed Up and Go Test; FRT - Functional Reach Test; ERSP - event-related spectral perturbations; RAGT - robotic-assisted gait therapy; VRRT - virtual reality reflection therapy; RIMT - Reinforcement-Induced Movement Therapy.

Author & year of publication	Rehabilitation outcome target	Intervention	Number of patients	Type of study	Result	Conclusion
Bortone et al. (2020) [25]	Upper extremity function in Cerebral Palsy	weaRable hAptic devices (VERA), cross over with manual therapy	8	Pilot cross over RCT	Improvement in upper extremity function in both control and experimental group	No statistically significant difference between both groups
Ögün et al. (2019) [26]	Post-stroke upper extremity function	Leap Motion-based 3D immersion VR	65	RCT	Improvement in upper extremity function	Statistically significant upper extremity motor improvement in the experimental group (p<0.05) assessed with the Fugl-Meyer Upper Extremity Scale
Choi et al. (2019) [27]	Post-stroke upper extremity function	Gesture recognition (GR) device mirror therapy	36	RCT	Improvement in upper extremity function	Statistically significant motor improvement in the GR group as compared to conventional mirror therapy group (p<0.05)
Oh et al. (2019) [28]	Post-stroke upper extremity function and cognitive function	VR + real instrument training	31	RCT	Improvement in upper extremity function	Statistically significant motor improvement in the experimental group for wrist extension (p< 0.04) and elbow flexion (p<0.022)
Feng et al. (2019) [29]	Parkinson’s disease balance and gait	VR training rehabilitation	28	RCT	Improved balance and gait	Statistically significant motor improvement in the experimental group (p < 0.05) across many scales: Berg Balance Scale (BBS), Functional Gait Assessment (FGA), and Timed Up and Go Test (TUG)
Park et al. (2019) [30]	Post-stroke upper extremity function	VR based planar motion exercise apparatus	26	Pilot RCT	Improvement in upper extremity function	Significant difference in motor function in both groups (p<0.05). Increased range of motion in the experimental group versus control for should abduction and internal rotation (non-statistically significant)
Lee et al. (2018) [31]	Post-stroke postural balance and upper extremity function	Game-based VR canoe paddling	30	RCT	Improvement in both balance and upper extremity function	Statistically significant motor improvement in upper extremity function and postural balance (p<0.05)
Karasu et al. (2018) [32]	Post-stroke static and dynamic balance	Nintendo Wii + conventional therapy	23	RCT	Improved in many categories	Motor improvement in both groups (non-statistically significant primary outcome), however group-time secondary outcome shows significant difference in the experimental group (p<0.001) for BBS and Functional Reach Test (FRT)
Bergmann et al. (2018) [33]	Post-stroke gait	VR augmented robotic-assisted gait therapy (RAGT)	20	Pilot RCT	Improved gait and motivation	Increased motivation in the experimental group, this group spent more time walking on the robot compared to the control group (<0.03). Statistically significant difference on the Functional Ambulation Classification (p<0.01) after intervention in both control and experimental group
Gandolfi et al. (2017) [34]	Parkinson’s disease balance and gait	Virtual Telerehabilitation using Nintendo Wii	76	RCT	Reduced postural instability	Statistically significant motor improvement in VR Telerehabilitation group compared to in-clinic rehabilitation group (p=0.04) on Berg Balance Scale
Calabrò et al. (2017) [35]	Post-stroke gait and balance	RAGT + VR (2D animated avatar) interaction using EEG oscillations	24	Pilot RCT	Improvement in gait and balance	RAGT + VR (experimental group) showed higher event-related spectral perturbations (ERSP) in specific fronto-central cortical affected brain areas as compared to the control group
In et al. (2016) [16]	Post-stroke postural balance and gait	Virtual reality reflection therapy (VRRT)	25	RCT	Improvement in many categories	Statistically significant improvement (p<0.05) in VRRT group across various scales such as postural sway, Functional Reach Test, BBS, and TUG
Ballester et al. (2016) [36]	Post-stroke upper extremity function	Reinforcement-Induced Movement Therapy (RIMT)	18	RCT	Motor improvement with RIMT	Statistically significant motor improvement 12 weeks post-trial (p<0.05)

Discussion

VR applications have rising popularity in neurorehabilitation, particularly in post-stroke patients. Some of the benefits of VR rehabilitation include the ease and adaptability of the technology, a virtual environment created to mimic the real world, and the capability of receiving visual, tactile, and auditory feedback in real-time [28,30,37] Moreover, immersive VR consoles have the unique ability to stimulate near-life experiences that patients cannot otherwise achieve [26-27]. A study by Lee et al. using a game-based canoe paddling VR intervention has shown that VR applications increase participation, motivation, and compliance when it comes to rehabilitation [31]. One of the major successes of VR-based rehabilitation stems from active reinforcement. When VR is combined with conventional rehabilitation, immediate concrete feedback regarding patient performance is received [28,30,37]. It can be attested that the visual stimuli in VR applications play an important role when it comes to immediate feedback. This unique aspect of VR cultivates a sense of achievement in patients enabling a stronger motivation for rehabilitation-related tasks, which in turn leads to better therapeutic outcomes and recovery [30,37]. 

Neuroplasticity 

**Figure 2:**
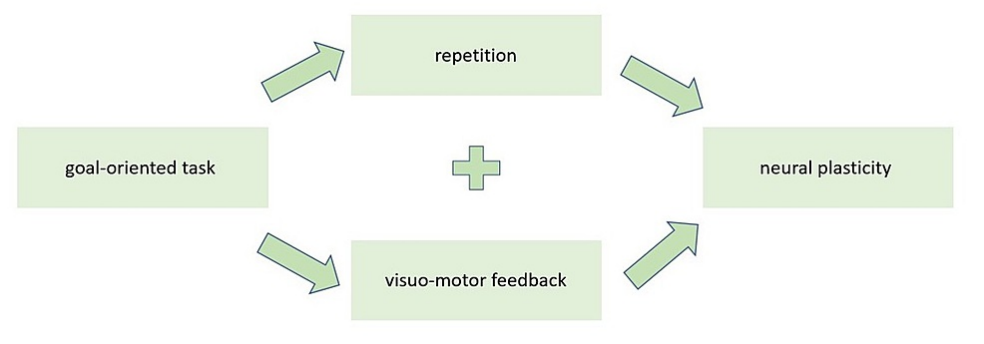
Factors involved in the remodeling of neural networks during neural plasticity

It has been suggested from clinical research that VR rehabilitation can enhance neuroplasticity [38]. A study by Ögün et al. using Leap Motion (Leap Motion, Inc., San Francisco, California) is one such example that suggests that motor movements in a VR environment stimulate plasticity [26]. The key elements in neural plasticity include task intensity and repetition [26,28,30,38]. Repetition of tasks is crucial when it comes to VR rehabilitation. An article by Oh et al. [28] discusses how adaptive plasticity occurs on more of a molecular level. During rehabilitation with a VR intervention, repetitive goal-oriented tasks are involved in the re-modeling of dendritic spines and thus the re-organization of movement representation in the motor cortex, premotor cortex, supplementary motor area, and the somatosensory cortex [28,38-41]. To add, it has been noted that multisensory perceptual feedback in VR rehabilitation has an impact on neural networking on the subcortical and cortical areas in the brain [28]. Furthermore, the study conducted by Ögün et al. suggests that with the evidence from neuroimaging methods, virtual motion can activate motion-related representation sites in the patient's brain, which supports the rewiring and cortical reorganization of the injured brain [26]. Put together; these studies suggest that task repetition combined with immediate multisensory feedback with re-training drives re-modeling on a neural level which in turn reflects changes in the representative cortexes through neural plasticity. Figure 2 illustrates this concept [26,28]

Motivation & Reinforcement in Upper Extremity Function 

Seven articles were identified in this systemic review which assessed upper extremity function using VR-based rehabilitation for patients diagnosed with stroke or cerebral palsy. After a stroke, upper extremity paresis is the most common motor deficit and is seen in approximately 80% of patients [26,42]. Patients who had a stroke are more likely to suppress the use of their paretic extremity; this is called "learned non-use" [36,43-44]. These patients oftentimes have a higher sensitivity to failure and success when it comes to motivation to use the affected arm [36]. This can lead to decreased motivation, compliance, and learned helplessness [36,43-44]. This is where the advantages of VR-based rehabilitation can be seen. Motivation and compliance can be achieved through active reinforcement and goal achievement. 

A study by Ballester et al. not only examined motor function with VR-based applications but also assessed a psychological component, more specifically, the reinforcement aspects of VR rehabilitation. The researchers proposed a Reinforcement-Induced Movement Therapy (RIMT). The concept of RIMT is to restore motor function capabilities by increasing the frequency of use in the affected arm through amplified goal-oriented tasks. With the design of this study, the experimental group had the opportunity to receive visuomotor feedback for the required tasks [36]. Real-time feedback was an important component of this study. 

In terms of methods, both the control and intervention groups underwent therapy thirty minutes per day, every day for six weeks [36]. This study design supports the need for repetition when it comes to effective synaptic plasticity. In this study, RIMT therapy showed statistically significant results (p<0.05) when it comes to upper extremity motor function for the VR exposure treatment group compared to controls, especially twelve weeks post clinical trial. In other words, VR rehabilitation not only had short-term therapeutic effects but these outcomes were seen three months post-study as well. Various scales can be used to assess upper extremity motor strength; in this study, the Fugyl-Meyer upper extremity scale was used [36]. 

Sustained Post-Trial Upper Extremity Motor Recovery 

In addition to Ballester et al.’s study, another study that also used the Fugyl-Meyer scale is Ögün et al.’s study using 3D Leap Motion [26]. One of the recent findings is the fact that the increased motor function seen after therapy with VR-based rehabilitation extends even past the treatment phase. In addition to Ballester et al.’s study, both Ögün et al.'s and Oh et al.’s articles demonstrate this finding [26,28,36]. The study by Ögün et al. used an immersive VR console that provided 360-degree interactive sessions. One of the key differences in this study is the use of immersive VR as opposed to regular VR. Through Leap Motion technology, investigators are provided real-time hand and trackable arm movements. The abundant repetitive tasks in this study (sixty-minute sessions, three days a week for six weeks), along with the capability of patients visualizing their avatar, allows for more fun and rewarding rehabilitation experience. This study further validates the concept of repetition in neural plasticity. To add, this study showed that after six weeks of VR rehabilitation using Leap Motion technology, patients demonstrated increased upper extremity motor function (p<0.05) [26]. 

When it comes to the sample size, Ögün et al.’s study had 65 patients who showed a significant statistical effect when compared to Ballester et al.’s study, which had 18 patients also showing a statistical effect [26,36]. The interesting point to note is that in Ballester et al.’s study, even though the sample size was smaller, a statistically significant effect on upper extremity motor function three months post-study was only seen in the group that underwent VR therapy compared to controls [36]. Sustained improvements in motor function seen three months post-trial is a very favorable finding in Ballester et al.’s study. This suggests that more clinical trials should be conducted in the future with larger sample sizes to examine the relationship between VR facilitated rehabilitation and long-term motor function recovery. 

The article by Oh et al. also evaluated upper extremity motor function in patients post-trial, more specifically one-month post-trial. Some of the differences in this study are that the study examines the effectiveness of VR-based rehabilitation in patients that are six months post-stroke and have also previously undergone post-stroke inpatient rehabilitation. This article is unique as the researchers set an inclusion criterion for patients with a Fugyl-Meyer upper-extremity scale score >18, which indicates anywhere from a mild to moderate limitation [28]. Interestingly, these researchers not only wanted to study if VR-based rehabilitation affected motor function recovery but also to what extent since they already had a baseline score from previous inpatient conventional rehabilitation. 

This study utilized VR in combination with real instrument training with objects such as doorbells, steering wheels, buttons, to name a few. The design of this study allows for enhanced fine motor use. In this study, statistical significance was particularly noted with the VR training group when it came to fine motor skills such as wrist extension (p< 0.04) and elbow flexion (p<0.022), and this improvement was sustained four weeks post-trial [28]. Since this study started with patients that already demonstrated a mild to moderate limitation, statistically significant improvements in fine motor skills are an important finding, especially since this was sustained one month post-trial. This study, like the previous studies above, also emphasized the importance of task repetition in motor recovery. The participants in this study underwent therapy for thirty minutes per day, three days a week for six weeks [28]. 

Mirror Neuron System & Virtual Reality 

One of the well-known concepts for the effectiveness of VR-based rehabilitation is within the Mirror Neuron System (MNS) [27]. This type of application tricks the mind into believing that the affected limb is moving. Two studies in the review have used variations of the mirror neuron concept together with VR applications to assess motor function in post-stroke patients [27,16]. A study by Choi et al. provides a concise explanation of the mirror neuron concept: a visual illusion that the paralyzed extremity is moving can be elicited with mirror therapy [27]. 

A mirror is placed at the center of the patient's body, covering the paralyzed and painful affected extremity. Once movement occurs on the unaffected limb, it appears that the paralyzed limb is moving through the reflection on the mirror [27]. In this study, three groups were randomized: standard mirror therapy, Gesture Recognition (GR) which is a form of mirror therapy through a 3D motion-input device, and the control group. The results of Choi et al.’s study favored an increased upper extremity motor function in the experimental group with VR + GR as compared to standard mirror therapy and the control group [27]. To add, a study by In et al. used a modified form of mirror therapy to assess lower extremity function. In's study utilized a virtual reality reflection therapy to assess improvement in balance for patients diagnosed with stroke [16]. The results of this study showed an increase in balance as assessed using the Berg Balance Scale [16]. To summarize, these two studies suggest that modifications of the classic mirror therapy using VR interventions show favorable outcomes for both upper and lower extremity motor function recovery. Interestingly the classic concept of mirror neuron-based applications has been applied to prospective research studies as well, particularly with the use of robotic-assisted gait therapy. 

Robot-Assisted Gait Training 

Recent advances in clinical trials have looked at VR-enhanced robot-assisted gait training (RAGT) [33,35]. Two pilot randomized controlled clinical trials in this review strived to assess the effectiveness of this novel approach. Both Bergman et al.’s study and Calabrò et al.’s study used a VR-augmented RAGT as their intervention [33,35]. The sample sizes in both studies were small (20 and 24 patients, respectively), but this is understandable since they are pilot trials. Even with a small sample size, both trials showed improved gait in the VR intervention group as compared to controls [33,35]. 

The study by Calabrò et al. used VR-enhanced RAGT through the use of an animated 2D avatar with a Lokomat (Hocoma, Zurich, Switzerland) for the RAGT apparatus [35]. The unique clinical explorations in this study were to visualize EEG activity during motor activities. The results of this study showed increased activation of the associated visual areas, precuneus, and premotor cortex in the RAGT + VR group as compared to the control group [35]. 

VR applications can entrain many brain areas that are involved in motor learning and planning [28,38-41]. A similar observation was noted in terms of feedback, through the real-time feedback received in VR, an increased cortical activation within the fronto-parietooccipital areas, especially within the MNS. As observed in the study by Bergman et al., the important thing to note is that use-dependent plasticity observed in the sensory-motor cortex can enhance motor recovery [35]. Furthermore, this study mentions that the use of an avatar can strengthen use-dependent plastic modifications within the sensory-motor regions of the brain that are part of the MNS [35,45-47]. With regards to the concept of neural plasticity, the use of a 2D avatar in this study reinforces the importance of real-time visual feedback as a key component in effective synaptic plasticity. This suggests one possible reason for how VR-based rehabilitation is more effective than conventional rehabilitation when it comes to motor function recovery. 

Interestingly, as opposed to Calabrò et al.’s study, which focused more on the neurological representation of VR-based rehabilitation in brain sites, the study by Bergman et al. assessed patient motivation. More specifically, motivation for various tasks was assessed using the Intrinsic Motivation Inventory (IMI). As predicted, a high level of motivation was seen throughout the entire duration for the experimental group. The intervention group, as compared to controls, spent a greater amount of time walking on the robot (p< 0.03) [33]. This study suggests that with this study design, direct feedback regarding a patient's performance was received through the interaction between actions in a VR scenario with motor behavior. 

Nintendo Wii Based VR Rehabilitation Post-Stroke 

As opposed to a more complex RAGT, a simpler and more convenient VR application is a Nintendo Wii console. Two principal investigators (Lee et al. and Karasu et al.) in this review utilized Nintendo Wii to assess motor function in stroke patients [31-32]. Lee et al.’s study used a game-based VR rehabilitation method to evaluate upper extremity function and postural balance. More specifically, Lee's study utilized a Nintendo Wii console for game-based canoe paddling. In this unique study, patients had access to the Nintendo Wii Sports Resorts game [31], and a canoe paddling apparatus was created to simulate the side-to-side swaying seen in this sport [31]. The study by Karasu et al. also utilized Nintendo Wii as a VR application to assess postural balance and gait in post-stroke patients. In this study, weight-shifting activities in Nintendo Wii were used to enhance postural stability [32]. Lee et al.’s study utilized a Manual Function Test to assess upper extremity function; for postural balance, a modified functional reach test (mFRT) was used [31]. In contrast, the Berg Balance Scale was used in Karasu et al.’s study to assess postural balance [32]. Interestingly, both studies incorporated the Nintendo Wii balance board for postural sway. 

The results of Lee et al.’s study show that the experimental group had a greater improvement that was statistically significant compared to the control group for upper extremity function. These researchers have suggested that the usage of the proximal part of a patient's upper extremity had a large impact on increased upper extremity motor function during the paddling tasks. To add, statistically significant improvements in postural balance were also seen after game-based VR rehabilitation sessions [31]. 

The researchers in Lee et al.’s study have suggested that the core muscles play a crucial role when it comes to stability. This muscle group helps connect the upper limbs to the lower limbs, and through this study, have discovered that through paddling exercises, increased trunk stability had a positive role in improving upper extremity function [31]. This study design indicates how VR applications can allow patients to immerse in as much real-life-like experience as possible, making the experience entertaining and rewarding. 

The ease and versatility of using Nintendo Wii are being seen more commonly in newer research studies. Karasu et al.’s study also showed statistically significant improvements in postural sway that are present after treatment and also extends into post-treatment (follow-up period) [32]. It is interesting to note that not only is Nintendo Wii being used to assess postural balance in post-stroke patients but also patients diagnosed with Parkinson's' disease. 

VR Rehabilitation in Parkinson's Disease 

**Figure 3:**
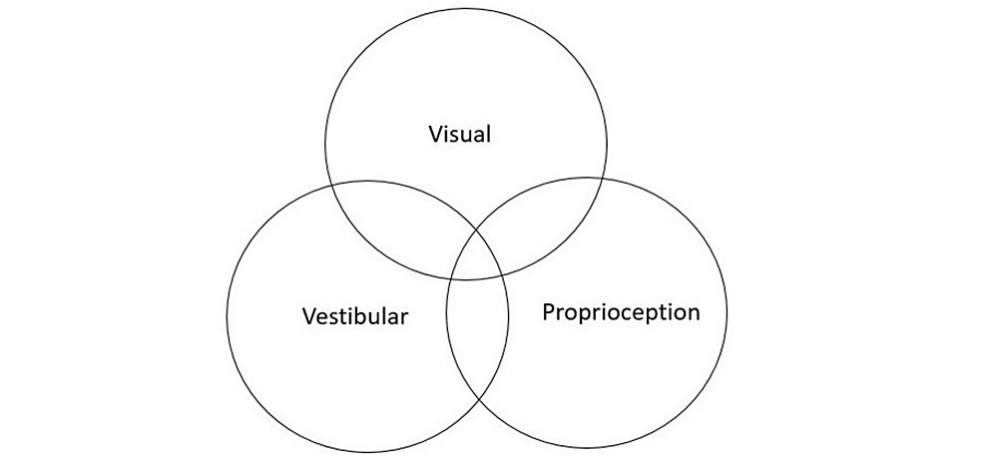
Interrelated systems for posture maintenance

Many studies have assessed the effectiveness of VR in post-stroke patients. Out of curiosity, can a similar principle be applied to other motor disorders such as Parkinson's disease? To answer this question, this review also looked for randomized controlled trials performed on patients diagnosed with Parkinson's disease as this illness also affects gait. Parkinson's disease is a movement disorder characterized by inadequate communication between the visual, vestibular, and proprioceptive systems. One of the deficits seen most often in patients is posture. Maintenance of posture also requires the interaction between the visual, vestibular, and proprioceptive systems [29,48]. Figure 3 illustrates the factors involved in the maintenance of posture. 

A study by Gandolfi et al. utilized Nintendo Wii-based game therapy for patients diagnosed with Parkinson's disease [34]. One of the most unique aspects of this study is that the researchers explored telerehabilitation. In this study, rehabilitation was done via physiotherapist-patient videoconferencing through Tele Wii [34]. There aren't many studies that have examined the effectiveness of home-based VR rehabilitation for postural stability improvements, especially in Parkinson's disease patients. This study aimed to compare the therapeutic outcomes of Nintendo Wii Fit-based exergame telerehabilitation to in-clinic sensory integration balance training. Gandolfi et al.’s study was a large study using 76 patients diagnosed with Parkinson's disease [34]. 

Similar to Karasu et al.’s study, the Berg Balance scale was used to assess effectiveness in the study conducted by Gandolfi et al. [32,34]. Significant (p=0.04) improvement in posture was seen in the group that underwent VR-based telerehabilitation. An important point to note is that VR-based rehabilitation has shown to be effective both in the clinic and via videoconferencing, which further enhances the ease and adaptability of this technology [34]. Furthermore, this study had a large sample size (76 patients) which illustrates that VR-based rehabilitation can be generalized to therapeutic advantages in Parkinson's disease as well, and a significant effect is not only seen in patients post-stroke. Since VR-based telerehabilitation is fairly new, more clinical trials would need to be conducted to support the implementation of this tool for remote rehabilitation. 

In addition, Feng and researchers assessed VR applications for gait and balance in patients with Parkinson's disease and have also shown statistically significant improvements in the experimental group. Once again, the concept of task repetition for neural plasticity has been illustrated clearly in this study. Feng et al.’s trial consisted of forty-five-minute sessions, five days a week for twelve weeks [29]. Out of all the articles discussed so far, this study seemed to have the longest duration. This can further suggest that task repetition for neural plasticity is not only effective in post-stroke patients but also in patients diagnosed with Parkinson's disease. 

Upper Extremity Function in Cerebral Palsy 

We have seen favorable results in motor recovery for VR-based rehabilitation in adult patients post-stroke and also for patients diagnosed with Parkinson's disease; however, is the similar effect seen in pediatric patients? One question that came up is how effective VR rehabilitation is when it comes to motor function recovery in pediatric patients? An article by Bartone et al. consisted of a pilot randomized controlled study that examined the effects of VR-based therapy on pediatric patients diagnosed with either cerebral palsy or developmental dyspraxia [25]. 

The study design consisted of two sessions per week for a total of eight weeks and not exceeding a maximum of fifteen minutes per session. This study utilized a VERA (weaRable hAptic) device along with an immersive virtual environment. Study participants had the opportunity to engage in a multi-sensory serious gaming experience. When evaluating upper extremity motor function, an improvement was seen in both experimental (VR enhanced therapy) and control groups (conventional rehabilitation therapy) with no statistical significance between the two [25]. One limitation to this study is a relatively small sample size (8 patients) which is understandable as a pilot randomized controlled trial. 

What we can take from this is that more clinical trials with pediatric patients undergoing VR-based therapies for rehabilitation should be conducted with larger sample sizes. One of the challenges that can be seen in future prospective trials is that the motivation and compliance for the entire study are more difficult to facilitate in younger children. 

Limitations

The articles that were chosen as part of this review underwent a rigorous risk of bias assessment. There are still some limitations. First of all, the types of articles that were included were limited to the English language and published within the last five years. This suggests that some high-quality studies may have been missed due to the inclusion criteria. Furthermore, this review included only articles that had free access, which may have limited the number of relevant articles. Finally, only randomized controlled trials were used, other forms of research were excluded. A few pilot randomized trials were also included with relatively small sample sizes to illustrate prospective research themes and to explore new topics in the field. 

## Conclusions

This systematic review was conducted to assess if a VR-based rehabilitation would be more effective than conventional rehabilitation therapy in improving motor function, particularly in patients that have a diagnosis of cerebral palsy, Parkinson's disease, or post-stroke. Through this analysis, we found that the implementation of VR in rehabilitation shows favorable results in motor function recovery, especially in the upper extremities and lower extremities for balance, gait, and posture. The way that VR can be used in multiple different conditions suggests the effectiveness and versatility of the application. An interesting find is in the variety of VR applications - from complex RAGT to simpler Nintendo Wii-based therapy. In addition, the inclusion of telerehabilitation shows prospects in the field. Furthermore, pilot trials with RAGT showing statistically significant results suggest that a definitive randomized controlled trial will be needed to further validate this concept. Lastly, it would be nice to see prospective clinical trials that aim to assess various VR-based applications in pediatric patients as well as there are many pediatric medical conditions in which VR-based therapy if effective in this population can aid with a more rapid therapeutic recovery.

## References

[1] Riva G, Wiederhold BK, Mantovani F (2019). Neuroscience of virtual reality: from virtual exposure to embodied medicine. Cyberpsychol Behav Soc Netw.

[2] Vincelli F, Riva G (2000). Virtual reality as a new imaginative tool in psychotherapy. Stud Health Technol Inform.

[3] Riva G, Molinari E, Vincelli F (2002). Interaction and presence in the clinical relationship: virtual reality (VR) as communicative medium between patient and therapist. IEEE Trans Inf Technol Biomed.

[4] Cipresso P, Giglioli IA, Raya MA, Riva G (2018). The past, present, and future of virtual and augmented reality research: a network and cluster analysis of the literature. Front Psychol.

[5] Sutherland IE (1965). The Ultimate Display. Multimedia: From Wagner to Virtual Reality.

[6] Biocca F (1997). The cyborg’s dilemma: progressive embodiment in virtual environments. J Comput Mediat Commun.

[7] Lombard M, Ditton T (1997). At the heart of it all: the concept of presence. J Comput Mediat Commun.

[8] Loomis JM, Blascovich JJ, Beall AC (1999). Immersive virtual environment technology as a basic research tool in psychology. Behav Res Methods Instrum Comput.

[9] Heeter C (2000). Interactivity in the context of designed experiences. J Interact Adv.

[10] Biocca F, Harms C, Gregg J (2001). The networked minds measure of social presence: pilot test of the factor structure and concurrent validity. 4th Annual International Workshop on Presence.

[11] Bailenson JN, Yee N, Merget D, Schroeder R (2006). The effect of behavioral realism and form realism of real-time avatar faces on verbal disclosure, nonverbal disclosure, emotion recognition, and copresence in dyadic interaction. Presence.

[12] Skalski P, Tamborini R (2007). The role of social presence in interactive agent-based persuasion. Media Psychol.

[13] Andersen SM, Thorpe JS (2009). An IF-THEN theory of personality: significant others and the relational self. J. Res. Pers.

[14] Slater M (2009). Place illusion and plausibility can lead to realistic behaviour in immersive virtual environments. Philos Trans R Soc Lond B Biol Sci.

[15] Sundar SS, Xu Q, Bellur S (2010). Designing interactivity in media interfaces: a communications perspective. Proceedings of the SIGCHI Conference on Human Factors in Computing Systems.

[16] In T, Lee K, Song C (2016). Virtual Reality Reflection Therapy improves balance and gait in patients with chronic stroke: randomized controlled trials. Med Sci Monit.

[17] Rutkowski S, Rutkowska A, Kiper P (2020). Virtual reality rehabilitation in patients with chronic obstructive pulmonary disease: a randomized controlled trial. Int J Chron Obstruct Pulmon Dis.

[18] da Silva Ribeiro NM, Ferraz DD, Pedreira É (2015). Virtual rehabilitation via Nintendo Wii® and conventional physical therapy effectively treat post-stroke hemiparetic patients. Top Stroke Rehabil.

[19] Oliveira MC, Bezerra LO, Melo Ângelo PH (2020). Game therapy a new approach to treat women facing mixed urinary incontinence: a study protocol. Neurourol Urodyn.

[20] Kiely DJ, Gotlieb WH, Lau S (2015). Virtual reality robotic surgery simulation curriculum to teach robotic suturing: a randomized controlled trial. J Robot Surg.

[21] Page MJ, McKenzie JE, Bossuyt PM (2021). The PRISMA 2020 statement: an updated guideline for reporting systematic reviews. BMJ.

[22] (2021). PubMed. https://pubmed.ncbi.nlm.nih.gov/.

[23] (2021). ScienceDirect. https://www.sciencedirect.com/.

[24] (2021). RoB 2: a revised tool for assessing risk of bias in randomised trials. BMJ.

[25] Bortone I, Barsotti M, Leonardis D, Crecchi A, Tozzini A, Bonfiglio L, Frisoli A (2020). Immersive virtual environments and wearable haptic devices in rehabilitation of children with neuromotor impairments: a single-blind randomized controlled crossover pilot study. J Neuroeng Rehabil.

[26] Ögün MN, Kurul R, Yaşar MF, Turkoglu SA, Avci Ş, Yildiz N (2019). Effect of Leap Motion-based 3D immersive virtual reality usage on upper extremity function in ischemic stroke patients. Arq Neuropsiquiatr.

[27] Choi HS, Shin WS, Bang DH (2019). Mirror therapy using Gesture Recognition for upper limb function, neck discomfort, and quality of life after chronic stroke: a single-blind randomized controlled trial. Med Sci Monit.

[28] Oh YB, Kim GW, Han KS, Won YH, Park SH, Seo JH, Ko MH (2019). Efficacy of virtual reality combined with real instrument training for patients with stroke: a randomized controlled trial. Arch Phys Med Rehabil.

[29] Feng H, Li C, Liu J (2019). Virtual reality rehabilitation versus conventional physical therapy for improving balance and gait in Parkinson's disease patients: a randomized controlled trial. Med Sci Monit.

[30] Park M, Ko MH, Oh SW (2019). Effects of virtual reality-based planar motion exercises on upper extremity function, range of motion, and health-related quality of life: a multicenter, single-blinded, randomized, controlled pilot study. J Neuroeng Rehabil.

[31] Lee MM, Lee KJ, Song CH (2018). Game-based virtual reality canoe paddling training to improve postural balance and upper extremity function: a preliminary randomized controlled study of 30 patients with subacute stroke. Med Sci Monit.

[32] Karasu AU, Batur EB, Karataş GK (2018). Effectiveness of Wii-based rehabilitation in stroke: a randomized controlled study. J Rehabil Med.

[33] Bergmann J, Krewer C, Bauer P, Koenig A, Riener R, Müller F (2018). Virtual reality to augment robot-assisted gait training in non-ambulatory patients with a subacute stroke: a pilot randomized controlled trial. Eur J Phys Rehabil Med.

[34] Gandolfi M, Geroin C, Dimitrova E (2017). Virtual reality telerehabilitation for postural instability in Parkinson's disease: a multicenter, single-blind, randomized, controlled trial. Biomed Res Int.

[35] Calabrò RS, Naro A, Russo M (2017). The role of virtual reality in improving motor performance as revealed by EEG: a randomized clinical trial. J Neuroeng Rehabil.

[36] Ballester BR, Maier M, San Segundo Mozo RM, Castañeda V, Duff A, Verschure PFMJ (2016). Counteracting learned non-use in chronic stroke patients with reinforcement-induced movement therapy. J Neuroeng Rehabil.

[37] Sveistrup H, McComas J, Thornton M (2003). Experimental studies of virtual reality-delivered compared to conventional exercise programs for rehabilitation. Cyberpsychol Behav.

[38] Kleim JA, Jones TA (2008). Principles of experience-dependent neural plasticity: implications for rehabilitation after brain damage. J Speech Lang Hear Res.

[39] Nudo RJ (2003). Adaptive plasticity in motor cortex: implications for rehabilitation after brain injury. J Rehabil Med.

[40] Hallett M (2005). Neuroplasticity and rehabilitation. J Rehabil Res Dev.

[41] Jang SH, You SH, Hallett M (2005). Cortical reorganization and associated functional motor recovery after virtual reality in patients with chronic stroke: an experimenter-blind preliminary study. Arch Phys Med Rehabil.

[42] Cramer SC, Nelles G, Benson RR (1997). A functional MRI study of subjects recovered from hemiparetic stroke. Stroke.

[43] Andrews K, Stewart J (1979). Stroke recovery: he can but does he?. Rheumatol Rehabil.

[44] Taub E, Uswatte G, Mark VW, Morris DM (2006). The learned nonuse phenomenon: implications for rehabilitation. Europa Medicophysica.

[45] Fadiga L, Craighero L (2004). Electrophysiology of action representation. J Clin Neurophysiol.

[46] Buccino G, Binkofski F, Fink GR (2001). Action observation activates premotor and parietal areas in a somatotopic manner: an fMRI study. Eur J Neurosci.

[47] Ramachandran VS, Hirstein W (1998). The perception of phantom limbs. The D. O. Hebb lecture. Brain.

[48] Lord S, Godfrey A, Galna B, Mhiripiri D, Burn D, Rochester L (2013). Ambulatory activity in incident Parkinson's: more than meets the eye?. J Neurol.

